# Mechanical strength and flexibility in $\alpha '$-4H borophene

**DOI:** 10.1038/s41598-021-87246-3

**Published:** 2021-04-06

**Authors:** Shobair Mohammadi Mozvashi, Mohammad Ali Mohebpour, Sahar Izadi Vishkayi, Meysam Bagheri Tagani

**Affiliations:** 1 Computational Nanophysics Laboratory (CNL), Department of Physics, University of Guilan, P. O. Box 41335-1914, Rasht, Iran; 2 School of Physics, Institute for Research in Fundamental Sciences (IPM), P. O. Box 19395-5531, Tehran, Iran

**Keywords:** Structural materials, Nanoscale materials, Structural properties, Two-dimensional materials

## Abstract

Very recently, a novel phase of hydrogenated borophene, namely $\alpha '$-4H, has been synthesized in a free-standing form. Unlike pure borophenes, this phase shows very good stability in the air environment and possesses semiconducting characteristics. Because of the interesting stiffness and flexibility of borophenes, herein, we systematically studied the mechanical properties of this novel hydrogenated phase. Our results show that the monolayer is stiffer (Y$_\text {xy}$ = $\sim$195 N/m) than group IV and V 2D materials and even than MoS$_2$, while it is softer than graphene. Moreover, similar to other phases of borophene, the inherent anisotropy of the pure monolayer increases with hydrogenation. The monolayer can bear biaxial, armchair, and zigzag strains up to 16, 10, and 14% with ideal strengths of approximately 14, 9, and 12 N/m, respectively. More interestingly, it can remain semiconductor under this range of tension. These outstanding results suggest that the $\alpha '$-4H is a promising candidate for flexible nanoelectronics.

## Introduction

Two-dimensional (2D) boron sheets (i.e., borophenes) have received considerable attention for the applications in high-speed and flexible nano-devices^1–4^. They have unusual properties such as anisotropic electronic structure, mechanical compliance, ultrahigh thermal conductance, optical transparency, and superconductivity^5–7^. Borophenes have outstanding mechanical properties, such as high ideal strengths and mild in-plane stiffness which carries potential use of them in making composites and electronic devices^8,9^. Moreover, they are the lightest 2D materials to date^10^ and also possess good flexibility which endows their potentials as flexible and wearable devices, biomedicines, and biosensors^8,11^. Besides, rather all borophenes have inherent anisotropy in most of the physical aspects, such as mechanical, electronic, and optical properties^10^.

Because of the rich bonding configuration of boron, borophene can be found in many phases and morphologies. The difference is mostly in the vacancy concentration, which strongly controls the stability of the phases. Up to now, many different phases of borophene, namely $\alpha$, $\beta$, $\gamma$, $\delta$, $\chi$, and $\psi$ have been theoretically predicted^12–14^ and a few of them have been synthesized on different substrates such as Ag (111), Au (111), Cu (111), and Al (111)^15–19^. However, separation of borophene from the substrate is a big challenge. As it is known, free-standing borophenes, more or less, possess partially occupied p$_z$ orbitals, which leads to unsaturated $\sigma$ and $\pi$ bonds. As a result, they are mostly unstable in the air environment and have metallic nature which confines their outstanding applications in nanoelectronics^20,21^.

Chemical functionalization is reported to be a feasible way for stabilizing borophene and other unstable 2D structures because it fills the partially occupied states and fixes the unsaturated bonds^22–27^. Among those, hydrogenation is regarded as the most effective strategy for stabilizing borophene and tuning its electronic and mechanical properties. Ultrahigh Fermi velocity, anisotropic thermal conductance, outstanding storage capacity, anisotropic electrical conductance, and magnetism have been reported for the hydrogenated borophenes and its nanoribbons^28–35^. These properties are strongly influenced by the hydrogen coverage, although the hydrogenation process could be a big challenge.

Very recently, a new phase of hydrogenated borophene ($\alpha '$-4H) was synthesized by in-situ thermal decomposition of sodium borohydride (NaBH$_4$) powders in a hydrogen-rich environment^36^. Unlike the other synthesized samples, this phase was grown without any metal substrate while showing ultrahigh stability under the air condition at temperatures up to 400 $^\circ$C. It was reported very stable in strong acid and base solvents. Surprisingly, $\alpha '$-4H borophene is a semiconductor with an indirect band gap of 2.48 eV which is very desirable for nanoelectronics and optoelectronics. Due to the facile and possible synthesis of $\alpha '$-4H borophene at a large scale and for its tremendous potential in electronic devices, in the first step, the mechanical properties of this material should be evaluated.

Mechanical properties are evaluated by the response of a crystal to the applied strain. Also, strain engineering is known as a common and important approach for tuning the electronic, magnetic, and other intrinsic properties of materials^37–44^. Therefore, the sustainability of a material against the applied strain should be known as well. A comprehensive understanding of mechanical properties of materials not only offers new interesting concepts to study, but also provides conditions for manufacturing innovative devices. It is important due to the need for materials with various strength, stiffness, and elasticity.

As we know, the interactive relation between theoretical and experimental investigations are very vital in discovery and tuning the properties of novel materials on demand. Herein, we systematically investigated the mechanical properties of the recently synthesized $\alpha '$-4H borophene using first-principles calculations. We firstly introduce the newly hydrogenated borophene and speak about its physical configuration, electronic structure, and dynamical stability, and then focus on the mechanical aspects and report its stiffness and strength. Meanwhile, we compare the differences between other pure and hydrogenated phases of borophene and discuss how the hydrogenation process can manipulate the material into a more desirable phase. Our results suggest that the newly synthesized $\alpha '$-4H borophene is a very interesting and promising material for flexible electronic applications.

## Computational details

We carried out first-principles calculations based on the density functional theory (DFT) as implemented in the Quantum ESPRESSO package^45^. The norm-conserving Vanderbilt pseudopotentials were employed for the description of electron-ion interaction. The PBE form of generalized gradient approximation (GGA) was chosen for the exchange-correlation functional. The DFT-D2 correction of Grimme was also employed to check if there is any difference in the structural results. The Brillouin zone was sampled by 20$\times$20$\times$1 k-points in the Monkhorst-Pack grid^46^. The kinetic energy cutoff was set to 50 Ry. To avoid spurious interactions, a vacuum space of 20 Åwas kept along the z-direction. All the atoms were fully relaxed until the Hellmann-Feynman force on each atom was less than 10$^{-4}$ eVÅ$^{-1}$.

The hydrogenation energy was calculated through:

1 $$\begin{aligned} E_{Hyd.}=\frac{E_{sheet}-(E_{pure}+4E_{H})}{12} , \end{aligned}$$

where $E_{sheet}$, $E_{pure}$, and $E_H$ are the total energy of hydrogenated borophene, pure borophene, and isolated hydrogen atom with consideration of spin polarization, respectively. Moreover, the cohesive energy was calculated through:

2 $$\begin{aligned} E_{c}=\frac{E_{sheet}-(8E_B+4E_H)}{12} , \end{aligned}$$

where $E_{sheet}$, $E_B$, and $E_H$ are the total energy of the hydrogenated borophene sheet, isolated boron and hydrogen atoms with consideration of spin polarization, respectively.

To check the dynamical stability, the phonon dispersion spectrum was calculated using the finite displacement method as implemented in the PHONOPY package^47^. The harmonic interatomic force constants (IFCs) were obtained using a 3$\times$3$\times$1 supercell containing 108 atoms and a 4$\times$4$\times$1 grid for k-point. Here, the convergence criterion of energy was set to 10$^{-8}$ eV.

The stress tensor is explained in Eq. S1 of the Electronic Supporting Information (ESI). Stress is directly obtained through S$_{11}$ and S$_{22}$ elements for the armchair and zigzag strains, respectively. However, for biaxial strains, the mean square value of biaxial stress was obtained by $\sqrt{(S_{11}^2+S_{22}^2)/2}$. Moreover, the obtained stress values were multiplied into the vacuum distance (2 nm) to get the unit of N/m.

## Results and discussion

### Structural and electronic properties

The freestanding hydrogenated borophene was synthesized recently by Hou et al.^36^. DFT simulations were also done by the authors for better understanding of the experiment. Several models were investigated and compared, eg. $\alpha '$-2H, $\alpha '$-4H, $\alpha '$-6H, etc. The preferred model was reported to be the $\alpha '$-4H, which is the nearest to the experiment. This configuration has the lowest formation energy and is the only semiconductor model. Therefore, we only focus on the $\alpha '$-4H phase which probably is the actual synthesized borophene.

**Figure 1:**
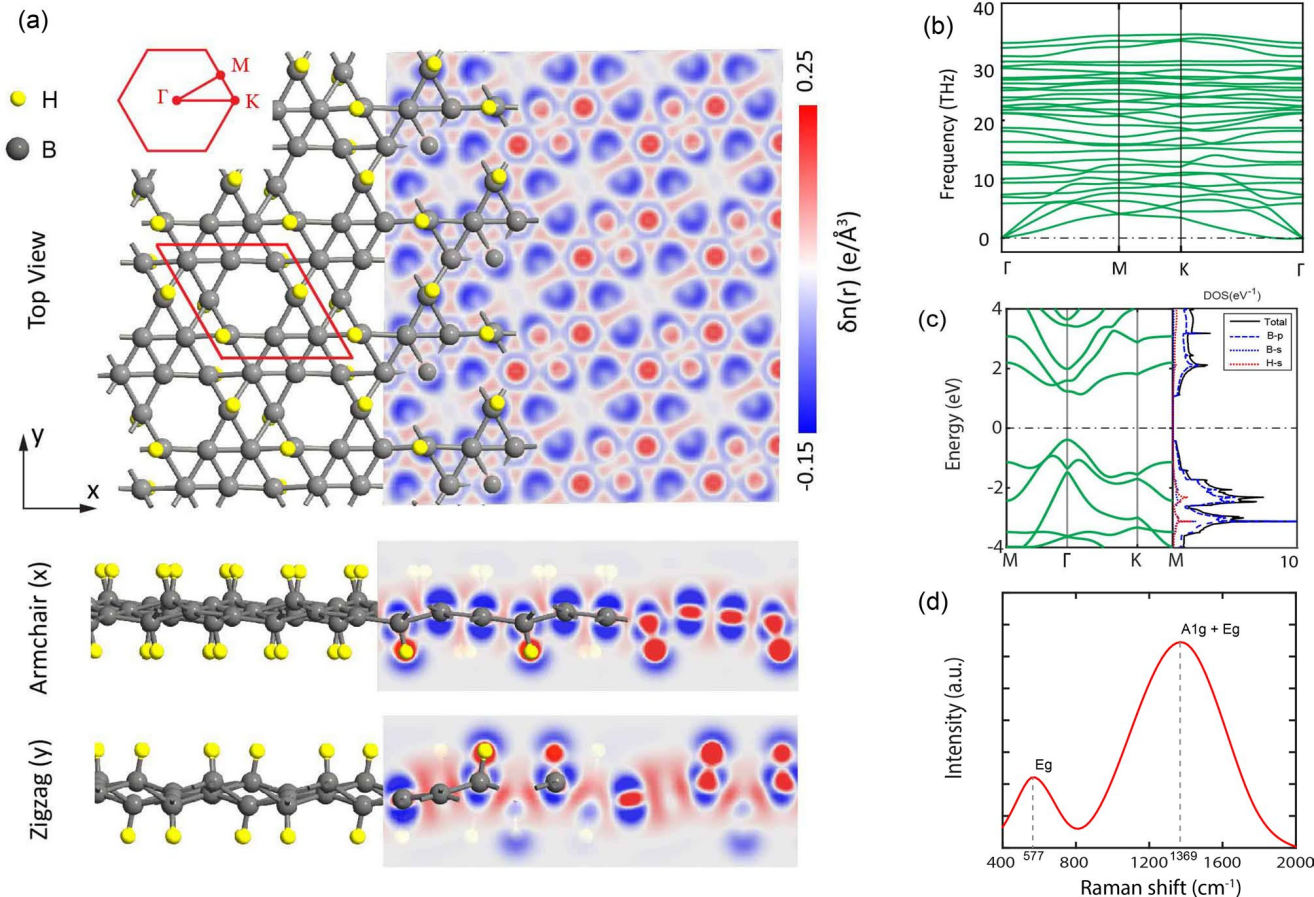
(**a**) Top and side views of the $\alpha '$-4H borophene together with electron difference density map. The x and y axes correspond to the armchair and zigzag directions, respectively. The unit cell and corresponding Brillouin zone are also shown. (**b**) Phonon dispersion spectrum of the $\alpha '$-4H borophene. (**c**) Electronic band structure and density of states (DOS) of the $\alpha '$-4H borophene. (**d**) Raman spectrum of the $\alpha '$-4H borophene.

Different positions for hydrogen atoms were tested and the optimized configuration, which is the most supportive one with the experiment in aspects of lattice constant, band gap, and dynamical stability was chosen. Figure 1a indicates the top and side views of the optimized $\alpha '$-4H borophene, where the unit cell is marked with a red rhombus. As can be seen, twelve atoms with a ratio of 2:1 (B:H) form the $\alpha '$-4H borophene, with a vacancy concentration of 1/9. The relaxed lattice constant was calculated to be 5.05 Å, which is in good agreement with the previous study^36^. The $\alpha '$-4H borophene has a buckled structure with a buckling height predicted to be 0.88 Å. Also, the bond lengths were obtained 1.69 and 1.77 Åalong the armchair and zigzag directions, respectively.

By comparing these bond lengths with the covalent radius of boron (0.88 Å)^48^, it is found that the armchair and zigzag directions are governed by covalent bonding. However, the armchair bonds have penetrated deeper into the covalent distance, therefore we expect a higher stiffness for the this direction. For the pure $\alpha '$ borophene, the bond lengths were calculated to be 1.68 Åfor both directions. Therefore, the adsorption of hydrogen atoms is seen to be responsible for the highly anisotropic behavior of $\alpha '$-4H borophene. The anisotropy is similarly observed from the electron difference density map in Fig. 1a, showing different accumulation (red) and depletion (blue) patterns. It is found that the maximum accumulation (0.25 eÅ$^{-3}$) is around hydrogen atoms, acting as electron acceptors. On the other hand, the maximum depletion (−0.15 eÅ$^{-3}$) is around five-coordinated boron atoms (bonded to hydrogen atoms), behaving as electron donors.

**Table 1:** Structural parameters of the pure and hydrogenated borophene at the GGA level: lattice constant (a), buckling height ($\Delta$), bond length (R), adsorption length (h), hydrogenation energy ($E_{Hyd.}$), and cohesive energy ($E_c$).

	a (Å)	$\Delta$ (Å)	R (Å)	h (Å)	E$_{Hyd.}$ (eV/atom)	E$_c$ (eV/atom)
$\alpha '$	5.05	0.37	1.68	–	–	$-6.25$
$\alpha '$-4H	5.05	0.88	1.69/1.77	1.22	$-0.84$	$-6.74$

The structural parameters calculated with PBE functional are listed in Table 1 and the results with other methods (LDA, GGA-D2) are available in Table S1 (ESI). As presented in Table 1, the hydrogenation energy is $-0.84$ eV/atom, indicating the great tendency of the pure structure to adsorb hydrogen atoms. The cohesive energy was additionally calculated to be $-6.74$ eV/atom to confirm that $\alpha '$-4H borophene is structurally stable. This value is smaller than those of other phases, calculated with the same method, such as $\alpha$ ($-5.76$), $\alpha '$ ($-5.76$), $\beta$ ($-5.65$), $\delta _3$ ($-4.88$), $\beta _{12}$ ($-5.71$), and $\chi _3$ ($-5.72$ eV/atom)^16^, showing the higher stability of the considered model. We also calculated the phonon dispersion spectrum to check the dynamical stability of $\alpha '$-4H borophene, as presented in Fig. 1b. We see no imaginary phonon mode, therefore, the structure is fully stable. The overlapping of the acoustic and optical modes is attributed to the robust phonon-phonon scattering which may result in low thermal conductivity. It is worth mentioning that the structural and dynamical stabilities of pure $\alpha '$ borophene were already validated by Wu et al.^12^

The band structure of $\alpha '$-4H borophene was calculated and presented in Fig. 1c. As can be seen, $\alpha '$-4H borophene is a semiconductor with an indirect band gap of 1.50 eV, where the valence band maximum (VBM) is located at the $\Gamma$ point, while the conduction band maximum (CBM) is in the $\Gamma$-K path. The VBM is well dispersed, signifying free holes, while the CBM is much flatter and non-parabolic, suggesting heavy electrons. In more detail, the calculated effective masses for electrons (at the CBM) are 0.25 $m_e$ and 0.49 $m_e$ along the armchair and zigzag directions, respectively, while the effective mass for holes (at the VBM) is 0.22 $m_e$ for both directions. This electron-hole asymmetry leads to the anisotropy of carrier mobility for p- and n-type doping.

Due to the underestimation of the band gap with the PBE functional, we also utilized the PBE0 hybrid functional, which is achieved by incorporating 25% of Hartree-Fock exchange with 75% of the PBE exchange. Using PBE0, the band gap is predicted to be 2.54 eV, which is in better agreement with the experiment (2.48 eV)^12^. To see the role of different orbitals in band edges, we calculated the total and partial density of states (DOS) and depicted them in Fig. 1c. As can be seen, both VBM and CBM are contributed by the p orbitals of boron, although, there is a week hybridization between s and p orbitals.

Figure 1d presents the Raman spectrum of $\alpha '$-4H borophene. We can see two peaks at 577 and 1369 cm$^{-1}$, which are representatives of *Eg* and $A1g+Eg$ vibrational modes of B-B cluster. These results are very supportive to the experimental Raman peaks at 743 and 1035 cm$^{-1}$^36^.

Overall, our structural results are in great agreement with the experiment^36^, so we can confidently rely and go on with the proposed model and the computation methods.

### Mechanical properties

To extract information about the stiffness and strength of a crystal, the variation of stress, lattice constants, and total energy should be evaluated under different strains. As it is known, a biaxial strain is defined as $\varepsilon =(a-a_0)/a_0$, where *a* and $a_0$ are the loaded and relaxed lattice constants, respectively. For uniaxial strains, a rectangular supercell with lattice constants of $2\mathbf {a}+\mathbf {b}$ and $\mathbf {b}$ containing 24 atoms was used. The uniaxial strain was applied to one direction (e.g. armchair) while the other side (e.g. zigzag) and also atoms positions were allowed to relax. We firstly report our final results as presented in Table 2 and subsequently discuss each one point by point.

**Table 2:** Mechanical parameters of the $\alpha '$-4H borophene: Young’s modulus (Y), Poisson’s ratio ($\nu$), critical strain ($\varepsilon ^*$), and ideal strength ($\sigma ^*$).

	Y (N/m)	$\nu$	$\varepsilon ^*$ (%)	$\sigma ^*$ (N/m)
Biaxial	195.56	–	16	14.06
Armchair (x)	164.54	0.191	10	8.99
Zigzag (y)	143.05	0.163	14	12.01

**Figure 2:**
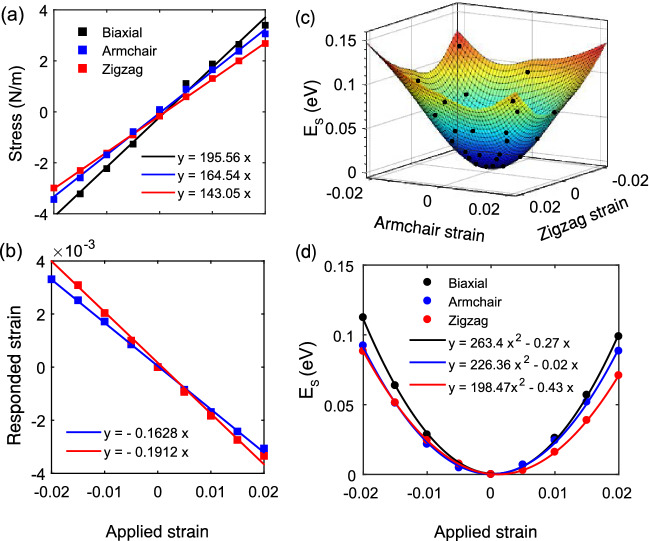
(**a**) Stress–strain curve in the harmonic region ($-2$% to 2%), used to calculate Young’s modulus. (**b**) Variation of responded (transverse) strain with the applied (axial) strain, used to calculate Poisson’s ratio. (**c**) Energy-strain 3D surface fitted by the actual energy of applied biaxial and uniaxial strains. (**d**) Energy-strain curve in the harmonic region, connected by polynomial fitted functions used to calculate stiffness constants.

Young’s modulus is calculated through:

3 $$\begin{aligned} Y_i= \frac{\partial \sigma _i}{\partial \varepsilon _i}, \end{aligned}$$

where $\sigma _i$ and $\varepsilon _i$ are the stress and strain in direction *i*. We define $Y_{xy}$ as the biaxial Young’s modulus, which is half of the shear modulus ($G_{xy}$). To calculate these moduli, we applied uniaxial and biaxial strains in the range of $-2$% to 2%, in steps of 0.5% and calculated the stress/strain relation as shown in Fig. 2a. As it is clear, the stress trend is rather linear in this range, therefore, it could be called the harmonic region. As mentioned in Table 2, the obtained Young’s moduli were 195.56, 164.54, and 143.05 N/m for biaxial, armchair, and zigzag strains, respectively.

Similar to those of other phases of borophene, these are very outstanding results that predict mild stiffness and flexibility for the monolayer at the same time. In other words, these moduli show that borophene is way stiffer than group IV and V elementals (Y = $\sim$ 23 to 60 N/m) and even MoS$_2$ (Y = $\sim$ 118 N/m), where it is more flexible than graphene (Y = $\sim$ 340 N/m)^49–53^. We can also see an anisotropic behavior as well, which is seen in most borophene phases^21^. This is due to the difference between bond lengths in the two directions, which has been already discussed in the structural section.

To have a better comparison, we explored Young’s modulus of other phases of borophene, especially the hydrogenated ones (borophanes). Moreover, to see the impact of hydrogenation on the mechanical properties, we re-calculated them for the pure $\alpha '$-borophene. This comparative information is listed in Tables S2 and S3 (ESI). The results show that the $\alpha '$-4H borophene is stiffer than T-borophane along the armchair and stiffer than C-borophene and B-borophene along the zigzag one^28,29^. Besides, it is more flexible than its mother pure phase and other pure borophenes as well. In other words, we can see that similar to other phases, the hydrogenation process decreases the stiffness and increases the anisotropy of the lattice. This may be raised from the breakage of solely sp$^2$ flat bonds in pure borophene and the emergence of buckled $\hbox {sp}^2 - \hbox {sp}^3$ bonds in hydrogenated ones. Moreover, as shown in Fig. 1a, the hydrogen atoms absorb much electron accumulation and weaken the strength and symmetry of the in-plane bonds.

To have more insight into the mechanical behavior of the monolayer, we also calculated the Poisson’s ratios and compared them with other phases of borophene. While applying uniaxial strain in one direction, an induced strain will be applied to the other (transverse) direction, spontaneously. Poisson’s ratio is the relation between these strains, which is achieved through:

4 $$\begin{aligned} \nu _{i}=-\frac{\partial \varepsilon _j}{\partial \varepsilon _i}, \end{aligned}$$

where $\varepsilon _i$ and $\varepsilon _j$ are the applied strain in direction *i* and the induced strain in direction *j*, respectively.

As presented in Fig. 2b and listed in Table 2, Poisson’s ratio of $\alpha '$-4H monolayer along the armchair ($\nu _x$) and zigzag ($\nu _y$) directions are 0.19 and 0.16, respectively. Similar to most of the 2D materials, especially borophenes, we can see a positive Poisson’s ratio in $\alpha '$-4H borophene. This means that the applied and the induced strains are against each other, as explained in Fig. S2. The results are also comparable with other phases, as presented in Table S2.

Again, we can see the mechanical anisotropy of $\alpha '$-4H borophene by the difference of Poisson’s ratio along the armchair and zigzag directions. Compared to other phases, as listed in Table S2, the anisotropy of $\alpha '$-4H borophene is more similar to W, B, and C-borophanes, and $\chi _3$ borophene, where Young’s moduli and Poisson’s ratios are higher along the armchair direction and lower along the zigzag one. On the contrary, in T-borophane and $\beta _{12}$-borophene, Young’s moduli and Poisson’s ratio are lower along the armchair and higher along the zigzag direction. The emergence of variant results between different phases of the same material are due to diverse structural configurations, which makes the study of borophenes very interesting and surprising.

Although Young’s moduli and Poisson’s ratio have already given sufficient perception about the mechanical properties of $\alpha '$-4H borophene, we can also go deeper into the problem by calculating the stiffness tensor. The stiffness tensor of a 2D system either can be obtained by Young’s moduli and Poisson’s ratio, based on Hook’s law^54^:

5 $$\begin{aligned} \varvec{\sigma _{2D}} = \begin{pmatrix} \sigma _{xx} \\ \sigma _{yy} \\ \sigma _{xy} \end{pmatrix} = \begin{pmatrix} Y_x &{} \nu _yY_x &{} 0 \\ \nu _xY_y &{} Y_y &{} 0 \\ 0 &{} 0 &{} G_{xy}(1-\nu _x\nu _y) \end{pmatrix} \begin{pmatrix} \varepsilon _{xx} \\ \varepsilon _{yy} \\ 2\varepsilon _{xy} \end{pmatrix} = \begin{pmatrix} C_{11} &{} C_{12} &{} 0 \\ C_{21} &{} C_{22} &{} 0 \\ 0 &{} 0 &{} C_{66} \end{pmatrix} \begin{pmatrix} \varepsilon _{xx} \\ \varepsilon _{yy} \\ 2\varepsilon _{xy} \end{pmatrix}, \end{aligned}$$

or can be extracted from the variation of strain energy, according to the analysis by Wei and Peng^55^:

6 $$\begin{aligned} C_{ij}=\frac{1}{A_0d_0}(\frac{\partial E^2_s}{\partial \varepsilon _i \partial \varepsilon _j}), \end{aligned}$$

where $A_0$ and $d_0$ are the relaxed unit cell area and the vacuum distance. Moreover, $E_s$ is the strain energy, given as $E_s=E_\varepsilon - E_0$, in which $E_\varepsilon$ and $E_0$ are total energy of the loaded and relaxed structures, respectively.

**Figure 3:**
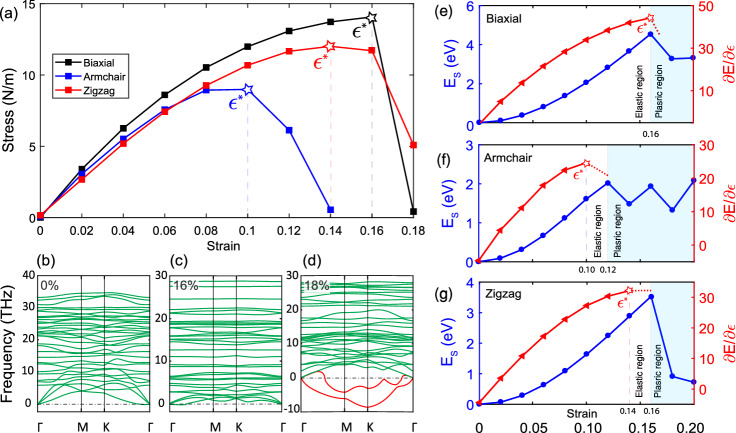
(**a**) Stress–strain curve in the range of 0 to 18% for obtaining the ideal strength. (**b**–**d**) Phonon band structure under different biaxial strains. (**e**–**g**) The energy/strain and energy-gradient/strain curves in the range of 0 to 20% used to calculate the traditional elastic and plastic regions for biaxial, armchair, and zigzag strains.

We first used the obtained Young’s moduli and Poisson ratios to calculate the stiffness tensor by use of Eq. (5). However, we also used the Wei-Peng method, Eq. (6), to compare the results. For the latter, we calculated the strain energy of the biaxial and uniaxial strains in the harmonic range and fitted them with polynomial functions, as shown in Fig. 2c, d. The results of the two procedures are fully supportive (< 1% difference), which are presented in Eq. (7):

7 $$\begin{aligned} {\varvec{C}}= \begin{pmatrix} 164.24 &{} 27.15 &{} 0 \\ 28.03 &{} 144.2 &{} 0 \\ 0 &{} 0 &{} 97.78 \end{pmatrix}. \end{aligned}$$

Because of the small Poisson’s ratios in $\alpha '$-4H borophene, the elastic constants are straightforwardly obtained by Young’s moduli. General speaking, we suggest that for 2D materials with small absolute Poisson’s ratio ($|\nu |<$ 0.2), the elastic constants can be simply obtained by:

8 $$\begin{aligned} C_{11}\simeq Y_x, \text { } C_{22}\simeq Y_y, \text { and } C_{66} \simeq (G_{xy}=Y_{xy}/2). \end{aligned}$$

After studying the elasticity and stiffness behavior of the monolayer in the harmonic region, we were keen to investigate its behavior in higher ranges of strains, so, its strengths and weaknesses under biaxial and uniaxial loadings be revealed. Ideal strength is defined by the maximum value of the stress/strain curve. To obtain this, we applied biaxial and uniaxial tensile strains to the monolayer and plotted the stress/strain curves in Fig. 3a. As shown in this figure and given in Table 2, the ideal strengths ($\sigma ^*$) are obtained as 14.97, 8.99, and 12.01 N/m under the corresponding critical strains ($\varepsilon ^*$) of 16%, 10%, and 14%, for biaxial, armchair, and zigzag directions, respectively. These critical strains are usually introduced as the strain of failure, say the ultimate strain in which the material can sustain and remains stable.

For assurance, we also calculated the phonon dispersion of the monolayer under different strains to see where the structure fails. As it is shown in Fig. 3b–d, the structure reserves its stability until the biaxial strain of 16%, and then it expresses a dynamical instability under 18%. This completely supports the results of the biaxial stress/strain curve.

For even more confirmation, we also plotted the curves of energy and energy-gradient ($\frac{\partial E}{\partial \varepsilon }$) by the applied strain which is shown in Fig. 3e–g. The energy/strain curves ascend like an upward parabolic until near the critical strains and after that they drop dramatically. In mechanical engineering, the area after the energy drop is usually called the plastic region (vs. elastic one), therefore, despite the dynamical instability, we also named it so. Moreover, the energy-gradient/strain curves also increase until the exact point of the critical strains and then drop (or softly decrease). Our results about the failure, by use of stress, phonon bands, and strain energy support each other very well.

Compared to the pure $\alpha '$ and other pure phases of borophene^8,56^, the hydrogenation leads to a very mild decrease in the ideal strength of the system. Moreover, the anisotropy between the x and y directions is increased as well. These effects are similar to the case of Young’s modulus, which could be explained again by the decreased in-plane electron distribution due to the hydrogenation. More importantly, the biaxial critical strain of $\alpha '$-4H borophene is higher than the pure $\alpha '$ and other pure phases of borophene, which means higher sustainability of the monolayer against tension and bend. This is obviously due to the lower Young’s moduli and higher flexibility of $\alpha '$-4H, compared to the pure borophenes.

**Figure 4:**
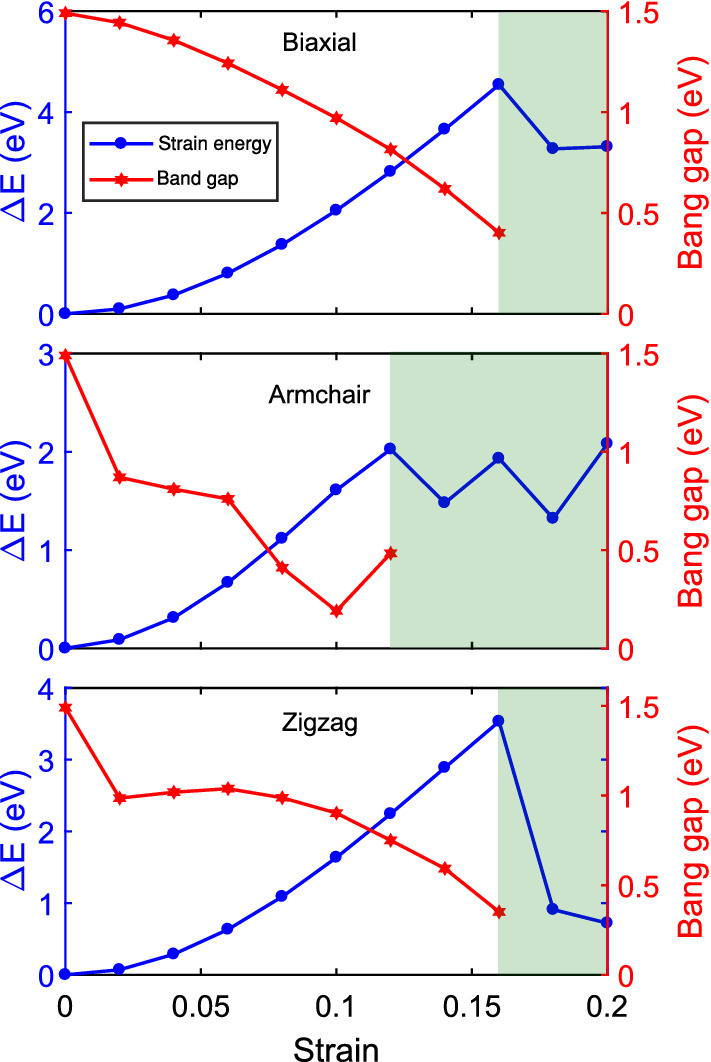
Variation of band gap with applied strains within the elastic region.

Due to its proper band gap and flexibility, as a suggestion, we emphasize the need for investigating the applications of $\alpha '$-4H borophene in flexible electronics. To ignite this, we calculated the variation of band gap with strain within the elastic region and present it to be a good end for this paper. As presented in Fig. 4, the band gap decreases mildly with biaxial strain. However, the reduction of the band gap possesses a more complicated trend with the uniaxial strains. Regardless of how the band gap behaves with different strains, the important point is that it only fluctuates within a narrow range and it never vanishes. In other words, as a potential flexible nano-device, the $\alpha '$-4H borophene remains semiconductor in case of applying any pressure less than the critical strain, which is very interesting for applied purposes.

## Conclusion

There are many predicted and synthesized phases of borophene, all of which possess outstanding properties that make the study of borophenes a very interesting subject in the field of 2D materials. However, the air-instability and metallic behavior are the drawbacks which confine their applications. The recently synthesized $\alpha '$-4H borophene is of the most importance, because of its super-stability and semiconducting behavior due to hydrogenation. Our results also accept these features. We continued the study of this phase in aspects of mechanical properties through first-principles calculations. We predict that the $\alpha '$-4H borophene is a flexible monolayer with sufficient but not too much stiffness and has good sustainability against tension. The most important point is that it can remain semi-conductor in the stable strain range which makes the monolayer a good candidate for flexible electronics and wearable devices.

## Supplementary Information

Supplementary Information 1.
