# Supplementary material for: Mechanical strength and flexibility in α′-4H borophene
Source: Sci Rep. 2021 Apr 6;11:7547. doi: 10.1038/s41598-021-87246-3 (PMC8024380; doi:10.1038/s41598-021-87246-3)
Supplement: Supplementary file 1 — Supplementary Information 1. [file 41598_2021_87246_MOESM1_ESM.pdf]

Electronic Supporting Information (ESI) for:
"Mechanical Strength and Flexibility in α'-4H Borophene".

Shobair Mohammadi Mozvashi[1], Mohammad Ali Mohebpour[1], Sahar Izadi Vishkayi[2], and Meysam Bagheri Tagani[1]

[1] Computational Nanophysics Laboratory (CNL), Department of physics, University of Guilan, P. O. Box 41335-1914, Rasht, Iran.
[2] School of Physics, Institute for Research in Fundamental Sciences (IPM), P. O. Box 19395-5531, Tehran, Iran.

Table S1: Structural parameters of the pure and hydrogenated borophene monolayer at different levels of theory: lattice constant (a), buckling height (Δ), average bond length (R), adsorption length (h), and cohesive energy (E_c).

		a (Å)	Δ (Å)	R (Å)	h (Å)	E_c (eV/atom)
α′	LDA	5.06	0.37	1.67	--	
	GGA.PBE	5.05	0.37	1.68	--	-6.25
	DFT-D2	5.05	0.37	1.68	--	-6.25
α′-4H	LDA	5.06	0.87	1.74	1.23	
	GGA.PBE	5.05	0.88	1.74	1.22	-6.74
	DFT-D2	5.03	0.89	1.74	1.22	-6.74

$$Stress\,Tensor = \begin{pmatrix} S_{11} & S_{12} & S_{13} \\ S_{21} & S_{22} & S_{23} \\ S_{31} & S_{32} & S_{33} \end{pmatrix} Ry/Bohr^3 \quad \text{(S1)}$$

Table S2: Mechanical parameters of different phases of borophene and borophane: Young moduli and Poisson's ratio along armchair and zigzag directions (x and y, respectively).

	Y_x (N/m)	Y_y (N/m)	ν_x	ν_y
**α'-borophene (here)**	**209.78**	**208.11**	**0.148**	**0.145**
**α'-4H-borophene (here)**	**164.54**	**143.05**	**0.191**	**0.163**
α-borophene [1]	210.56	210.56	0.196	0.196
β_{12}-borophene [1]	179.00	203.12	0.176	0.199
χ^3-borophene [1]	198.50	182.70	0.116	0.107
C-borophane [1]	172.24	110.59	0.177	0.144
B-borophane [2]	191.17	80.54	0.265	0.112
T-borophane [2]	124.59	148.16	0.179	0.212
W-borophane [2]	176.73	156.54	0.132	0.117

Table S3: Comparison between mechanical parameters of the pure α′ and hydrogenated α′-4H borophene: Young's modulus (Y), Poisson's ratio (ν), the critical strain (ε^*), and ideal strength (σ^*).

		Y (N/m)	ν	ε^* (%)	σ^* (N/m)
α′	Biaxial	251.33	---	14	18.01
	Armchair	209.78	0.148	12	13.23
	Zigzag	208.11	0.145	12	16.71
α′-4H	Biaxial	195.56	---	16	14.06
	Armchair	164.54	0.191	10	8.99
	Zigzag	143.05	0.163	14	12.01

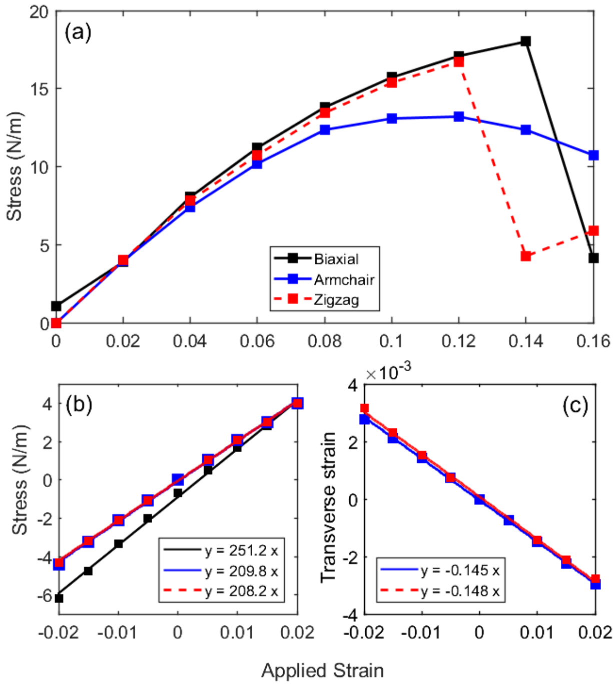

Figure S1. Mechanical properties of the pure B_8 monolayer: (a) Stress-strain curve in the range of 0 to 16% for obtaining the ideal strength. (b) The stress-strain curve in the harmonic region (-2% to 2%), used to calculate Young's modulus. (c) Variation of responded (transverse) strain with the applied (axial) strain, used to calculate Poisson's ratio.

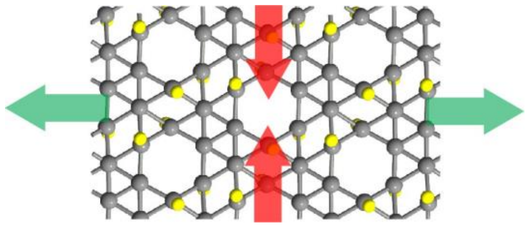

Figure S2: A schematic of a positive Poisson's ratio.

References

[1] Z. Wang, T.-Y. Lü, H.-Q. Wang, Y. P. Feng, and J.-C. Zheng, "High anisotropy of fully hydrogenated borophene," *PCCP*, vol. 18, no. 46, pp. 31424-31430, 2016.

[2] Z. Wang, T.-Y. Lü, H.-Q. Wang, Y. P. Feng, and J.-C. Zheng, "New crystal structure prediction of fully hydrogenated borophene by first principles calculations," *Sci Rep*, vol. 7, no. 1, pp. 1-11, 2017.
